# HIV stigma in Kenya: A family medicine led community orientated primary care approach

**DOI:** 10.4102/phcfm.v13i1.3054

**Published:** 2021-10-28

**Authors:** Peter M. Kioko, Maureen W. Kamau, Norah A. Obungu, Emma A. Khabure, Anne M. Simmelink, Katana Geoffrey, Fleur O. de Meijer

**Affiliations:** 1 Department of Family Medicine, Medical College of East Africa, Aga Khan University, Nairobi, Kenya; 2 Department of Health, Kilifi County, Kenya

**Keywords:** community oriented primary care (COPC), HIV/AIDS, family medicine, intervention, improvement, sensitisation, stigma

## Abstract

The Kenyan Ministry of Health envisages that family physicians should play an important role in the implementation of community orientated primary care (COPC) in collaboration with the community health team. The Kenyan Community Health Strategy forms a solid basis for the implementation of the COPC model. Residents and faculty of the Family Medicine department at the Aga Khan University Hospital Nairobi collaborated with the Kaloleni sub-county of Kilifi County government near Mombasa in a five-step COPC process to better understand and act against the high prevalence of HIV stigma in the coastal region. Firstly, a deeper understanding of human immunodeficiency virus (HIV) stigma was acquired through community visits and work in the comprehensive care clinic. Secondly, a collaborative implementation team was formed to design a targeted and feasible intervention. In a participatory approach, a two-step intervention was employed, firstly sensitising healthcare workers and community health volunteers (CHVs) on the high prevalence of HIV stigma in their community and educating them on HIV-related issues. Secondly, the information was disseminated to the community through home visits by CHVs, health talks and the set-up of an HIV support group at the facility. This short report illustrates the important contribution of family physicians to implementation of COPC and capacity building of the primary healthcare team.

## Community orientated primary care and the Kenya Community Health Strategy

**FIGURE 1:**
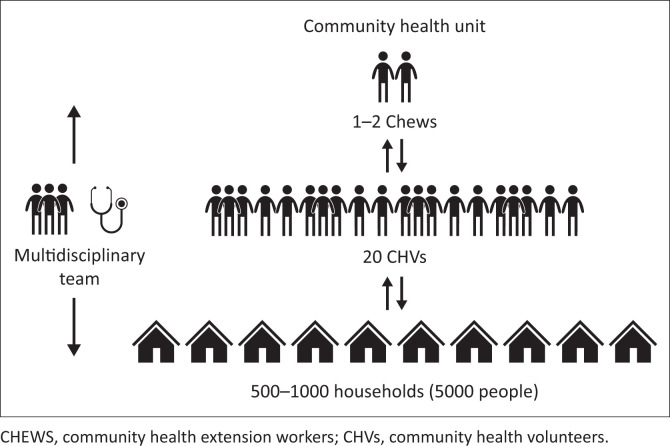
Structure of a Kenyan Community Health Unit^2^ (2020–2025). The multidisciplinary team consists of nurses, clinical officers, public health officers and a psychologist at ward level and a community strategy focal person and HIV/AIDS and sexually transmitted infections (STIs) coordinator at sub-county level.

Community orientated primary care (COPC) has been promoted in family medicine academic circles as an essential method for providing high-quality primary care, especially in underserved and rural areas. Within this model, health providers of different cadres work together with community members to promote health, prevent disease and support treatment based on their assessed health needs in a defined geographical area.^1^ The Kenyan Community Health Strategy 2020-2025 forms a basis for implementation of COPC. In this strategy, each sub-county is divided into administrative wards and within each ward there are several health facilities. A community health unit, linked to the first referral facility (usually a dispensary), consists of one or two community health extension workers (CHEWs) who oversee 20 community health volunteers (CHVs). Community health volunteers provide home-based services to 5000 people (500–1000 households) in the catchment area of the dispensary. The Community Health Unit is supported by a multidisciplinary team from the ward and sub-county and together they focus on local health challenges (Figure 1).^2^

### Family medicine community orientated primary care rotation

Since its inception in 2012, the Family Medicine Department at the Aga Khan University in Nairobi has collaborated with the Ministry of Health in Kilifi County to strengthen local healthcare systems whilst enhancing future family physician’s skills in COPC. In their first and third years of training, family medicine residents work for 6 weeks in primary healthcare facilities in Kaloleni sub-county, a rural area inland from Mombasa. Under direct supervision of family physicians and guided by an online module, residents learn to enhance the capacity and skills of rural health facility staff in clinical care. They also collaborate within the community health unit to complete a five-step COPC project as follows: first year of training: (1) identifying and characterising the community, (2) involving the community, (3) identifying the community health problems, third year of training: (4) developing an intervention and (5) implementing and evaluating the impact of the intervention. The 2019 cohort of family medicine residents together with the Ministry of Health in Kilifi County, endeavoured to address HIV stigma in a rural Kenyan community in a COPC project.

During the initial stages of the project, HIV-related stigma was identified as the focus for the COPC project through a prioritisation process.^3^ The project was undertaken in the Tsangatsini dispensary catchment area, a rural village in Kaloleni with an estimated population of 193 692 inhabitants.^4^ Tsangatsini dispensary is a level two health facility and offers services including maternity and child health services, laboratory services, pharmacy services and outpatient services including a comprehensive care clinic for people living with HIV. Family medicine residents undertook home visits and worked in the comprehensive care clinics to better understand the underlying reasons for HIV stigma and to enable a targeted intervention. It emerged that, apart from self-stigmatisation by patients living with HIV, there was insufficient knowledge of HIV amongst CHVs and healthcare workers (HCWs) who contributed to HIV stigma in the community. According to the HIV stigma index of 2014, Kilifi County had a stigma index of 48%, which is amongst the highest rates in the country. The stigma of having HIV negatively affects HIV care and follow up after diagnosis. It also impacts the quality of care given to HIV patients and isolates people in the community. Insufficient knowledge on HIV in Kilifi County can partially explain the relatively high HIV stigma index in comparison to other counties.^6^

## Intervention

**FIGURE 2:**
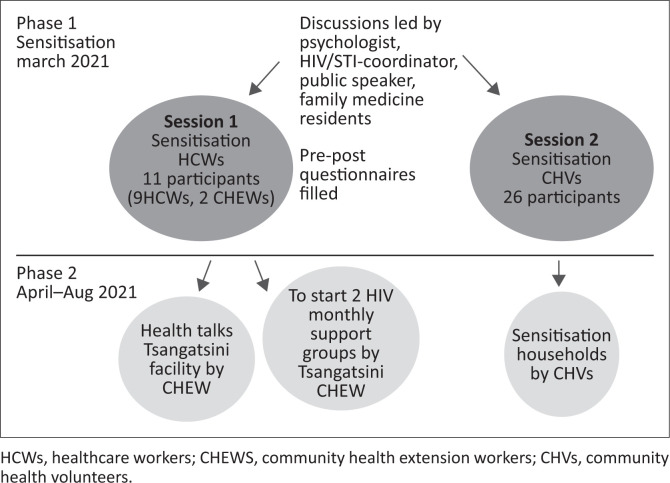
Intervention plan for HIV-stigma project.

Family medicine residents worked together with the facility and community CHEWs and members of the multidisciplinary team, namely the sub-county public health officer, community health strategy focal person, HIV/STI-coordinator, a psychologist and reproductive health nurse, to discuss the approach to HIV stigma in the area. A two-phased strategy (Figure 2) was employed, geared at increasing awareness amongst HCWs and CHVs that stigma exists in their facilities and communities, whilst increasing their knowledge on HIV and later disseminating this knowledge to the community.

Phase 1 consisted of HIV-related sensitisation exercises, which were seen as the most impactful and resource efficient intervention for the area. A total of 10 HCWs, 2 CHEWs and all CHVs attached to Tsangatsini Dispensary were invited for 2 separate sensitisation meetings. The intervention was a one-day exercise facilitated by a trained psychologist, a certified HIV/STI trainer who was a member of both the Kaloleni community and the four family medicine residents. A public speaker with a HIV-positive status was invited to further raise awareness on stigmatisation. The content of the presentation was tailored to the needs of the respective groups.

Phase 2 consisted of the development of an HIV knowledge dissemination plan in collaboration with the HCW in-charge of the Tsangatsini facility and the facility’s CHEWs. The following action plan was included:

- Weekly health education talks to patients in the facility on HIV and HIV stigma given by the trained facility CHEW
- Formation of two HIV support groups (one for children, one for adults) at Tsangatsini facility led by the CHEW
- Human immunodeficiency virus sensitisation activities by the CHVs during regular home visits. This involved discussing HIV-transmission with household members and informing them on the existence of HIV stigma and discrimination in the community.

In the first month 32 CHVs conducted 226 sensitisation home visits and gave one health talk on HIV and HIV stigma. Monthly HIV support groups had not started because of low clinic attendance by HIV-positive patients. This might have been caused by a regional increase in coronavirus disease 2019 (COVID-19) cases at the start of the roll-out of the project that inhibited HIV-infected patients from coming to the clinics. The facility manager however expected to be able to start both support groups in the month to come. Continued health talks at the facility and discussion on HIV during home-visits by CHVs during further roll-out of the project are expected to have a positive effect on HIV-related stigma.

## Suggestions for future research and conclusion

Through the COPC rotation, family medicine residents learn about working with a multidisciplinary team and encouraging effective participation of the community. This provides them with a vital opportunity to practice integrating primary care practice and public health. They also develop their leadership skills by developing an intervention for a locally identified health problem. Moreover, they are involved in training and capacity building, with additional obvious benefits to the local health team.

Human immunodeficiency virus stigma is an under-recognised health problem, which seriously hampers HIV care and increases the burden of the disease for those affected and their caregivers. More initiatives targeting HIV stigma in this area are therefore urgently needed. In addition, the impact of such projects should be monitored and evaluated. This is unfortunately not possible with the current format of the family medicine programme but ways of improving are currently explored.

In conclusion, this report presents an example of how the community can be involved in designing an intervention for a locally identified health problem, through the process of COPC under the guidance of family physicians, whilst at the same time providing a valuable learning experience for medicine residents.
